# Sleep-related hallucinations in patients with Parkinson’s disease

**DOI:** 10.1371/journal.pone.0276736

**Published:** 2022-10-25

**Authors:** Tomoko Komagamine, Keisuke Suzuki, Norito Kokubun, Junpei Komagamine, Akiko Kawasaki, Kei Funakoshi, Koichi Hirata

**Affiliations:** 1 Department of Neurology, Dokkyo Medical University, Tochigi, Japan; 2 Department of Internal Medicine, National Hospital Organization Tochigi Medical Center, Tochigi, Japan; Sapienza University of Rome: Universita degli Studi di Roma La Sapienza, ITALY

## Abstract

Given that sleep-wake cycle dysfunction can cause hallucinations in Parkinson’s disease patients, sleep-related hallucinations may be a different subtype from hallucinations that occur only during full wakefulness. However, few studies that distinguish the onset situations of hallucinations related to sleep from those that occur in full wakefulness have been conducted to investigate hallucinations in Parkinson’s disease patients. Therefore, we conducted a multicenter observational study to investigate the prevalence of and factors associated with sleep-related hallucinations in patients with Parkinson’s disease. Information on hallucinations was collected by using a questionnaire and face-to-face interviews. Of 100 consecutive patients with Parkinson’s disease, 29 (29%) reported sleep-related hallucinations, and 16 (16%) reported hallucinations only in the full wakefulness. A longer duration of Parkinson’s disease treatment (OR 1.35, 95% CI 1.07 to 1.72), higher Beck Depression Inventory-II scores (OR 1.07; 95% CI 1.01 to 1.14), and higher rapid eye movement sleep behavior disorder scores (OR 5.60; 95% CI 1.54 to 20.38) were independent factors associated with the presence of sleep-related hallucinations in a multivariable analysis. Sleep-related hallucinations, but not daytime hallucinations, were associated with probable rapid eye movement sleep behavior disorder. Phenomenological discrimination between sleep-related hallucinations and daytime hallucinations is important for elucidating the full pathology in Parkinson’s disease and the mechanisms underlying hallucinations.

## Introduction

Sleep-related hallucinations are defined by the American Academy of Sleep Medicine as vivid perceptual experiences that occur as a person falling asleep (hypnagogic) or waking up (hypnopompic) [1]. Based on the definition by the National Institute of Neurological Disorders and Stroke and National Institute of Mental Health, hallucinations in Parkinson’s disease (PD) are defined as abnormal perceptions without a physical stimulus that can involve any sensory modality [2]. Patients with PD frequently experience hallucinations with insights, including well-structured visual hallucinations and minor hallucinations, during the course of illness [3, 4]. While older age, longer disease duration and dopaminergic treatment were associated with the presence of hallucinations in patients with PD [5], early-stage and drug-naïve patients also often reported having hallucinations and hallucinatory phenomena [4, 6, 7].

Patients with PD often experience sleep disturbances, including insomnia, hypersomnia, rapid eye movement (REM) sleep behavior disorder (RBD), and excessive daytime sleepiness [8]. The presence of RBD in PD is related to visual hallucinations and cognitive impairment [9]. RBD is a risk factor for hallucinations in both narcolepsy and PD [10].

The possibility of REM intrusions into the waking state as a mechanism of hallucinations has been discussed [11, 12]. Sleep onset REM periods with polysomnography, which is one of the diagnostic signs of narcolepsy, are observed in PD patients with hallucinations [11]. Hypocretin/orexin neuronal loss occurs in PD as well as narcolepsy and increases disease progression in PD [13, 14]. These experimental and pathological results show that hallucinations in PD might reflect a narcolepsy-like REM sleep abnormality. However, in patients with PD, there have been a few studies on the prevalence of sleep-related hallucinations [10, 15], which are one of the clinical tetrads of narcolepsy.

To investigate sleep-related hallucinations and their related factors in PD patients, we conducted an observational study of the differences in clinical profiles between those with sleep-related hallucinations and those with hallucinations *only* occurring during daytime activities (daytime hallucinations). In addition, to compare the PD patients with controls in the prevalence of PD-like hallucinatory experiences, we also included healthy adults without neuropsychiatric diseases.

## Methods

### Study setting and design

A prospective observational study was conducted at Dokkyo Medical University Hospital, National Hospital Organization Tochigi Medical Center, Kanma Memorial Hospital, and Nasu Neurosurgical Hospital. These four hospitals are located in Tochigi Prefecture, near capital rural areas in Japan. Parkinson’s disease patients and adult controls were recruited from the outpatient clinic at the neurology departments of these hospitals. The protocol of the present study was approved by the Dokkyo Medical University Hospital Ethical Committee (No. 29001). All the present studies were performed in accordance with the Declaration of Helsinki.

### Participants

We recruited 108 consecutive PD patients who attended the outpatient clinics of the four hospitals from April 2017, to March 2019. All patients were diagnosed by neurologists in accordance with the Movement Disorder Society Clinical Diagnostic Criteria for Parkinson’s disease [16]. Patients who refused to answer all the elements of the questionnaire were excluded. After excluding eight patients, 100 patients with PD were included. We also recruited 60 age-matched family members of the patients as healthy controls. Controls who had a medical history of psychiatric/neurological diseases or regular visits to the hospital for sleep disorders were excluded. All the participants provided written informed consent.

### Data collection and outcome measures

Information on patient age, sex, onset age and duration of PD, and medications was collected by neurologists caring for the patients. We asked the patients and their caregivers to use the original screening questionnaire (see the S1 File) for the presence of hallucinations, the timing of the hallucinations (when wide awake during daytime activities and/or around sleep), the type of hallucinations (minor type, visual, auditory, tactile, olfactory), and the content of the hallucinations. After obtaining answers to the questionnaire, we conducted semistructured interviews with the patients and their caregivers, and the collected information included examples of hallucinations. Hallucinations were defined as feelings or perceptions without existence or physical stimuli that could involve any sensory modality [2].

Minor-type hallucinations included visual illusion and presence sense. Based on previous studies, visual illusions were defined as brief misperceptions of objects that differ from objective reality, and presence hallucinations were defined as vivid sensations that someone was present nearby in the absence of sensory clues [2, 4, 7].

For comparison, we performed the same screening questionnaire and interviewed healthy adults without neuropsychiatric manifestations and sleep disorders. Among healthy individuals, the term hallucination was also used for their abnormal perceptions without actual physical stimuli, based on the guidelines of previous publications [1, 17–19].

The timing of hallucinations was classified into the following two categories: hallucinations occurring only in the full wakefulness (daytime) and/or around sleep (sleep-related). Sleep-related hallucinations were defined as vivid perceptual experiences that occurred as a person falling asleep (hypnagogic) or waking up (hypnopompic) [1]. Sleep-related hallucinations or daytime hallucinations were distinguished by self-report in the questionnaire.

To evaluate sleep-related problems and depressive mood, all the participants were asked to complete the following questionnaires: a Japanese version of the REM sleep behavior disorder screening questionnaire (RBDSQ-J) [20], four essential features regarding restless legs syndrome [21], the Japanese Epworth Sleepiness Scale (JESS) [22], the Pittsburgh Sleep Quality Index (PSQI) [23], and the Beck Depression Inventory-II (BDI-II) [24]. In addition, Parkinson’s disease patients were asked to complete the Parkinson’s Disease Questionnaire-8 (PDQ-8) [25] and the Parkinson’s Disease Sleep Scale 2 (PDSS-2) [26] to evaluate subjective disability and sleep disturbance due to PD. If any questions were not answered, the examiner asked them to complete the items. For the assessment of cognitive dysfunction in PD patients, the revised Hasegawa Dementia Scale (HDS-R) was used [27]. Cognitive impairment was defined as a revised Hasegawa Dementia Scale score lower than 20. For adult controls, the presence of cognitive impairment was determined by history and behavior at an outpatient visit. The dataset is fully available in an attached S2 File.

### Statistical analysis

Statistical analysis was performed using Stata version 15 (LightStone, Tokyo, Japan). Descriptive statistics were used to describe the characteristics of the study population. The comparison between the PD patients and adult controls was performed by using chi-square tests for categorical variables and Mann–Whitney U tests for continuous variables. To investigate the differences in clinical characteristics between the participants who had sleep-related hallucinations and those who did not, chi-square tests and Mann–Whitney U tests were also used. In addition, we investigated the differences in clinical characteristics between PD patients who had sleep-related hallucinations and those who had daytime hallucinations by using these methods. To investigate the factors associated with the presence of sleep-related hallucinations among the PD patients, multivariate analysis with binary logistic regression was performed. The association between the presence of sleep-related hallucinations and the following variables was examined: age, sex, onset age, duration, Hoehn and Yahr stage of PD, dementia, restless leg syndrome, and JESS, PSQI, BDI, PDQ-8, PDSS-2, and RBD scores. Variables were removed one-by-one until all remaining variables had a *p* value of less than 0.2 by using a backward stepwise method. For these analyses, the level of significance was set at 5%.

## Results

### Characteristics of patients with Parkinson’s disease and controls

The patients with PD and controls were 71.6 (SD 10.0) and 70.7 (SD 11.8) years old on average, respectively (Table 1). The proportions of women were 51.0% and 65.0% in patients and controls, respectively. For the patients, the average onset age was 65.7 (SD 10.7) years old, and the mean Hoehn and Yahr stage was 2.8 (SD 1.1). The average levodopa dose was 284 (SD 183) mg. Twenty-three patients (23.0%) had cognitive impairment, and 12 (12.0%) were not receiving any antiparkinson medications at the time of the survey. Restless leg syndrome was found in 46% of the patient group and 18.3% of the control group.

**Table 1:** Characteristics of patients with Parkinson’s disease and adult controls.

	Patients with Parkinson’s disease (n = 100)	Adults without neuropsychiatric diseases (n = 60)	*p value* ^a^
Mean age, year (SD)	71.6 (10.0)	70.7 (11.8)	0.96
Female sex	51 (51.0)	39 (65.0)	0.08
Medical history			
Dementia	23 (23.0)	0 (0.0)	< 0.001
Restless leg syndrome	46 (46.0)	11 (18.3)	< 0.001
Mean onset age of PD, year (SD)	65.7 (10.7)	NA	NA
Mean duration of PD treatment	3.9 (4.0)	NA	NA
Hoehn and Yahr stage, mean (SD)	2.8 (1.1)	NA	NA
Mean dose of levodopa (SD)	284 (183)	NA	NA
Current use of dopamine agonists	47 (47.0)	NA	NA
The 8-item Parkinson’s disease questionnaire, mean (SD)	22.9 (18.2)	NA	NA
Parkinson’s disease sleep scale-2, mean (SD)	15.2 (9.3)	NA	NA
Japanese Epworth Sleepiness Score			
Mean, SD	7.4 (6.2)	3.5 (3.7)	< 0.001
More or 11 points	22 (22.0)	3 (5.0)	0.004
Pittsburgh Sleep Quality Index			
Mean, SD	6.4 (3.9)	4.5 (3.5)	< 0.001
More or 6 points	55 (55.0)	17 (28.3)	0.001
Beck depression inventory score			
Mean, SD	13.2 (10.5)	5.2 (6.4)	< 0.001
More or 11 points	53 (53.0)	10 (16.7)	< 0.001
RBD score			
Mean, SD	3.8 (2.5)	2.2 (2.0)	< 0.001
More or 5 points	33 (33.0)	7 (11.7)	0.003
Illusions	15 (15.0)	4 (6.7)	0.11
Any hallucinations	45 (45.0)	17 (28.3)	0.04
Timing of hallucinations			
Around sleep	29 (29.0)	9 (15.0)	0.04
Daytime	16 (16.0)	8 (13.3)	0.65
Type of hallucinations			
Minor type	28 (28.0)	10 (16.7)	0.10
Visual	30 (30.0)	8 (13.3)	0.02
Auditory	19 (19.0)	8 (13.3)	0.35
Tactile	7 (7.0)	1 (1.7)	0.13
Olfactory	6 (6.0)	0 (0.0)	0.05

^a^Comparisons between patients with Parkinson’s disease and controls were performed by using the chi-square test for categorical variables and the Mann–Whitney U test for continuous variables.

### Prevalence of hallucinations

Hallucinations were observed in 45% of the patients with PD and 18% of the healthy adult controls. Sleep-related hallucinations were more likely to be reported in the patients than in the controls (29.0% versus 15.0%, *p* = 0.04), while the proportion of participants who reported *only* daytime hallucinations was similar in the patients with PD and controls (16.0% and 13.3%, *p* = 0.65). In the PD group, the most common types of hallucinations were visual hallucinations (n = 30, 30%), followed by minor-type hallucinations (n = 28, 28%) and auditory hallucinations (n = 19, 19%). In the control group, minor hallucinations were most common (n = 10, 16.7%), followed by visual (n = 8, 13.3%) and auditory hallucinations (n = 8, 13.3%). No control participant reported an olfactory hallucination. The proportions of participants who reported visual hallucinations were significantly greater in the PD group than in the control group (*p* = 0.02). Although there was no statistically significant difference, all types of hallucinations other than visual hallucinations tended to be more frequently reported in the PD group than in the control group.

### Significant differences and factors associated with sleep-related hallucinations in Parkinson’s disease patients and adults without neuropsychiatric diseases

Of all the PD patients, 29 (29.0%) reported sleep-related hallucinations. Those who reported sleep-related hallucinations exhibited a higher proportion of restless leg syndrome, higher levodopa dose, longer duration of PD treatments, and higher Hoehn and Yahr stage than those of the PD patients who did not report them (PD without hallucinations and PD with only daytime hallucinations) (Table 2). PDQ-8 scores, PDSS-2 scores, JESS scores, PSQI scores, BDI-II scores, and RBD scores were also significantly higher in the PD patients who reported sleep-related hallucinations than in those who did not. Of all the healthy controls, 9 (15.0%) reported sleep-related hallucinations. The healthy adults who reported sleep-related hallucinations were older and more likely to have restless leg syndrome and depressed mood than those who did not.

**Table 2:** Characteristics of patients with Parkinson’s disease and controls with and without sleep-related hallucinations.

	Patients with Parkinson’s disease			Controls		
	Sleep-related hallucination			Sleep-related hallucination		
	Present (n = 29)	Absent (n = 71)	*p value* ^a^	Present (n = 9)	Absent (n = 51)	*p value* ^a^
Mean age, year (SD)	73.2 (7.6)	70.9 (10.8)	0.55	78.3 (11.7)	69.4 (11.5)	0.03
Female sex	16 (55.2)	35 (49.3)	0.59	6 (66.7)	33 (64.7)	0.91
Medical history						
Dementia	10 (34.5)	13 (18.3)	0.08	0 (0.0)	0 (0.0)	NA
Restless leg syndrome	19 (65.5)	27 (38.0)	0.01	5 (55.6)	6 (11.8)	0.002
Mean onset age of PD, year (SD)	63.8 (10.4)	66.5 (10.7)	0.20	NA	NA	NA
Mean duration of PD treatment	7.0 (5.0)	2.7 (2.8)	< 0.001	NA	NA	NA
Hoehn and Yahr stage, mean (SD)	3.2 (1.2)	2.6 (1.0)	0.03	NA	NA	NA
Mean dose of levodopa (SD)	393 (177)	239 (166)	< 0.001	NA	NA	NA
Current use of dopamine agonists	15 (51.7)	32 (45.1)	0.54	NA	NA	NA
The 8-item Parkinson’s disease questionnaire, mean (SD)	32.8 (21.4)	18.8 (15.1)	0.001	NA	NA	NA
Parkinson’s disease sleep scale-2, mean (SD)	21.5 (10.5)	12.6 (7.5)	< 0.001	NA	NA	NA
Japanese Epworth Sleepiness Score						
Mean, SD	10.1 (7.5)	6.4 (5.3)	0.02	5.7 (3.9)	3.1 (3.6)	0.06
More or 11 points	11 (37.9)	11 (15.5)	0.01	0 (0.0)	3 (5.9)	0.46
Pittsburgh Sleep Quality Index						
Mean, SD	7.8 (3.6)	5.9 (3.8)	0.01	3.3 (2.7)	4.7 (3.5)	0.28
More or 6 points	21 (72.4)	34 (47.9)	0.03	2 (22.2)	15 (29.4)	0.66
Beck depression inventory score						
Mean, SD	19.6 (10.3)	10.6 (9.5)	< 0.001	8.8 (7.9)	4.5 (5.9)	0.14
More or 11 points	22 (75.9)	31 (43.7)	0.003	4 (44.4)	6 (11.8)	0.03
RBD score						
Mean, SD	5.0 (2.4)	3.3 (2.4)	< 0.001	2.8 (2.9)	2.1 (1.9)	0.62
More or 5 points	17 (58.6)	16 (22.5)	< 0.001	2 (22.2)	5 (9.8)	0.28

^a^Comparisons between Parkinson’s disease patients with and without sleep-related hallucinations were performed by using the chi-square test for categorical variables and the Mann–Whitney U test for continuous variables.

In multivariate analysis, a longer duration of PD treatment (OR 1.35, 95% CI 1.07 to 1.72), higher BDI-II scores (OR 1.07; 95% CI 1.01 to 1.14), and higher RBD scores (OR 5.60; 95% CI 1.54 to 20.38) were the only independent factors associated with the presence of sleep-related hallucinations (Table 3).

**Table 3:** Results of multivariable analysis^a^ for factors associated with sleep-related hallucinations among patients with Parkinson’s disease.

	Odds ratio (95% confidence interval)	*p value*
Mean duration of treatment^b^	1.35 (1.07 to 1.72)	0.01
Mean dose of levodopa^b^	1.00 (1.00 to 1.01)	0.11
Current use of dopamine agonist	0.29 (0.07 to 1.28)	0.10
Beck depression inventory score^b^	1.07 (1.01 to 1.14)	0.03
REM sleep behavior disorder score of 5 or more	5.60 (1.54 to 20.38)	0.01
Restless leg syndrome	2.84 (0.80 to 10.09)	0.11

^a^Variables were removed one-by-one until all remaining variables had a *p* value of less than 0.2 by using a backward stepwise method.

^b^Continuous variable use.

### Comparison of characteristics between Parkinson’s disease patients with sleep-related hallucinations and those with only daytime hallucinations

Of all the PD patients, 16 (16.0%) reported having only daytime hallucinations. Those who indicated having only daytime hallucinations had a lower proportion of restless leg syndrome, shorter duration of PD treatment, lower dose of levodopa, lower PDSS-2 scores, and lower BDI-II scores than those of the PD patients who indicated having sleep-related hallucinations (Table 4). Tactile hallucinations were less likely to be reported in the patients with PD who indicated having only daytime hallucinations than in those who answered having sleep-related hallucinations (0.0% versus 24.1%, *p* = 0.03).

**Table 4:** Comparison of characteristics between Parkinson’s disease patients with sleep-related hallucinations and those with only daytime hallucinations.

	Sleep-related hallucinations (n = 29)	Only daytime hallucinations (n = 16)	*p value* ^a^
Mean age, year (SD)	73.2 (7.6)	74.4 (8.6)	0.05
Female sex (%)	16 (55.2)	5 (31.3)	0.12
Medical history			
Dementia	10 (34.5)	5 (31.3)	0.83
Restless leg syndrome	19 (65.5)	5 (31.3)	0.03
Mean onset age of Parkinson’s disease, year (SD)	63.8 (10.4)	70.3 (8.5)	0.004
Mean duration of Parkinson’s disease treatment	7.0 (5.0)	3.1 (2.8)	0.01
Hoehn and Yahr stage, mean (SD)	3.2 (1.2)	3.1 (1.2)	0.95
Mean dose of levodopa (SD)	393 (177)	225 (156)	0.003
Current use of dopamine agonists	15 (51.7)	8 (50.0)	0.91
The 8-item Parkinson’s disease questionnaire	32.8 (21.4)	23.3 (16.7)	0.12
Parkinson’s disease sleep scale-2	21.5 (10.5)	14.8 (7.5)	0.03
Japanese Epworth Sleepiness Score			
Mean, SD	10.1 (7.5)	8.9 (6.5)	0.73
More or 11 points	11 (37.9)	4 (25.0)	0.38
Pittsburgh Sleep Quality Index			
Mean, SD	7.8 (3.6)	6.5 (4.6)	0.17
More or 6 points	21 (72.4)	8 (50.0)	0.13
Beck depression inventory score			
Mean, SD	19.6 (10.3)	13.0 (6.7)	0.02
More or 11 points	22 (75.9)	11 (68.8)	0.61
RBD score			
Mean, SD	5.0 (2.4)	4.1 (2.9)	0.16
More or 5 points	17 (58.6)	6 (37.5)	0.17
Illusion	9 (31.0)	6 (37.5)	0.66
Type of hallucinations			
Minor type	21 (72.4)	7 (43.8)	0.06
Visual	20 (69.0)	10 (62.5)	0.66
Auditory	15 (51.7)	4 (25.0)	0.08
Tactile	7 (24.1)	0 (0.0)	0.03
Olfactory	2 (6.9)	4 (25.0)	0.09

^a^Comparisons between Parkinson’s disease patients with sleep-related hallucinations and only daytime hallucinations were performed by using the chi-square test for categorical variables and the Mann–Whitney U test for continuous variables.

### Comparison of characteristics between Parkinson’s disease patients with only daytime hallucinations and without hallucinations

The PD patients without hallucinations (n = 55) and PD patients with only daytime hallucinations (n = 16) did not differ in age, sex, onset age, treatment duration, levodopa dose, agonists, PDQ-8, PDSS-2, JESS, PSQI, or RBD score. (Table 5). Hoehn and Yahr stage (*p* = 0.03) and BDI scores (*p* = 0.03) were significantly higher among PD patients with only daytime hallucinations.

**Table 5:** Demographic and clinical features of Parkinson’s disease patients with only daytime hallucinations and without any hallucinations.

	No hallucination (n = 55)	Only daytime hallucinations (n = 16)	*p value* ^a^
Mean age, year (SD)	69.9 (11.2)	74.4 (8.6)	0.12
Female sex (%)	30 (54.6)	5 (31.3)	0.10
Medical history			
Dementia	8 (14.6)	5 (31.3)	0.13
Restless leg syndrome	22 (40.0)	5 (31.3)	0.53
Mean onset age of PD, year (SD)	65.4 (11.1)	70.3 (8.5)	0.10
Mean duration of PD treatment	2.6 (2.8)	3.1 (2.8)	0.49
Hoehn and Yahr stage, mean (SD)	2.4 (0.9)	3.1 (1.2)	0.03
Mean dose of levodopa (SD)	244 (170)	225 (156)	0.78
Current use of dopamine agonists	24 (43.6)	8 (50.0)	0.65
The 8-item Parkinson’s disease questionnaire	17.6 (14.5)	23.3 (16.7)	0.21
Parkinson’s disease sleep scale-2	12.0 (7.4)	14.8 (7.5)	0.18
Japanese Epworth Sleepiness Score			
Mean, SD	5.6 (4.7)	8.9 (6.5)	0.046
More or 11 points	7 (12.7)	4 (25.0)	0.23
Pittsburgh Sleep Quality Index			
Mean, SD	5.7 (3.6)	6.5 (4.6)	0.68
More or 6 points	26 (47.2)	8 (50.0)	0.85
Beck depression inventory score			
Mean, SD	9.9 (10.2)	13.0 (6.7)	0.03
More or 11 points	20 (36.4)	11 (68.8)	0.02
RBD score			
Mean, SD	3.1 (2.2)	4.1 (2.9)	0.21
More or 5 points	10 (18.2)	6 (37.5)	0.10

### Examples of hallucinated contents

The content of the hallucinations is summarized in Table 6. PD patients reported well-formed hallucinations and detailed experiences, including hallucinations involving dogs, cats, fish images, children with colorful dresses, musical hallucinations, and the feeling of moving spiders or mice moving under the clothes. Some PD patients and controls reported hallucinations involving deceased family or friends. One patient with PD answered whenever he hallucinated his deceased son came, and he asked his wife to fix a cup of tea for the son. A control participant answered that she could not see but sometimes felt the presence of her deceased friends, and she prayed for the world to be at peace. The controls experienced things that could not be explained well (figure, someone) and ambiguous sounds (murmurs, bubbles of voices). The control participants reported more vague experiences than PD patients.

**Table 6:** Descriptions of hallucinations in patients with Parkinson’s disease and controls.

	Patients with Parkinson’s disease		Adults without neuropsychiatric diseases	
	Sleep-related hallucinations	Daytime hallucinations	Sleep-related hallucinations	Daytime hallucinations
Minor type	Presence sense of children, stranger, deceased family	Presence sense/passing of children, stranger, deceased family	Presence sense of deceased family or friends	Presence sense of vague figure
Visual	People, children, animals, face, objects	People, children, animals, face, objects	Deceased family, figure	Figure
Auditory	Music, footsteps, instrument sounds	Instrument sounds, raindrops	Voice/footsteps of deceased family	Murmur, bubbles of voices, sounds
Tactile	Feelings of crawling spiders, running mice in underwear, licking by a dog, tickling by a child	-	Feeling pushed chest and floating	-
Olfactory	A delicious smell	Curry, flowers	-	-

## Discussion

In our study, sleep-related hallucinations in patients with PD were associated with a longer duration of PD treatment, depressed mood, and higher RBD scores, while daytime hallucinations were associated with PD severity and depressed mood. Previous studies have identified various factors that are associated with hallucinations in PD patients. However, most previous studies did not discriminate daytime hallucinations from sleep-related hallucinations [3, 5, 28–30]. Our results indicate that patients with sleep-related hallucinations might represent a specific subtype of PD, and the discrimination of these types of hallucinations would be necessary for the investigation of the relationship between hallucinations and PD.

RBD is the strongest risk factor for developing PD and is considered as a prodromal marker of PD [31]. Secondary RBD patients associated with brainstem lesions were identified, and the pathophysiology of RBD has been presented in relation to the Braak staging system for PD, that is, lesions spreading from the brainstem to the cortex [32]. Our findings indicate that sleep-related hallucinations in PD might reflect the spread of brainstem lesions.

RBD can occur after the onset of motor symptoms as well as before the onset of PD. In advanced stages of PD, RBD is more common [8]. When subtyping PD with non-motor symptoms, the RBD subtype includes visual hallucinations, color vision abnormalities, freezing, and falling [33]. Also, PD patients with probable RBD had higher rates of anxiety, depression, hallucinations and orthostatic hypotension than those without RBD [34]. Brain perfusion studies in isolated RBD have revealed increased perfusion in the pons, putamen and hippocampus and decreased perfusion in the frontal and temporoparietal cortices, parieto-occipital and limbic lobes and cerebellum [35]. In a brain network study, patients with PD and RBD showed more wider changes of nodal properties in functional networks than PD patients without RBD [36]. On the basis of these studies, compared with daytime hallucinations, sleep-related hallucinations in PD might originate from more extensive brain lesions.

RBD is a risk factor for hallucinations in both narcolepsy and PD [10]. Narcolepsy-like hypersomnia is also common in PD patients [37]. In addition, among PD patients with excessive daytime sleepiness, 39% had a narcolepsy-like phenotype (two or more sleep-onset REM periods during five sessions of the multiple sleep latency test) [38]. Previous studies have shown hypothalamic dysfunction in PD patients [39], and the loss of hypocretin/orexin cells in the hypothalamus increased with the progression of Parkinson’s disease [13, 14]. Because narcolepsy is caused by the dysfunction of hypocretin/orexin neurons in the lateral hypothalamus [40], a similar mechanism might underlie sleep-related hallucinations in PD patients.

Hallucinations in PD patients are not threatening experiences [29, 30], whereas hallucinations in those with narcolepsy and isolated sleep paralysis frequently elicit fear and other emotional responses [40, 41]. Dreams that occur during REM sleep also frequently provoke fear and anxiety [12]. The lack of fear associated with PD hallucinations might indicate that the dysfunction of the hypocretin/orexin system in PD does not activate the limbic system network involving the amygdala, unlike normal REM dreams and the dysfunction of hypocretin/orexin neurons in narcolepsy.

The hallucinations occurring in the two time periods exhibited different patterns in the sensory modality of the hallucinations. While olfactory hallucinations were uncommon around sleep, tactile hallucinations did not occur when wide awake. The results that tactile hallucinations occurred around sleep are consistent with those of previous reports of tactile hallucinations in patients with PD [42]. Visual hallucinations were the most common in patients with hallucinations that occurred in both time periods. In previous studies, visual, auditory, and tactile experiences were common among patients with narcolepsy and in healthy individuals during isolated sleep paralysis [10, 43]. The modalities of sleep-related hallucination in PD were also similar to those observed in narcolepsy and isolated sleep paralysis.

In our study, the prevalence of hallucinations in controls who did not have any neuropsychiatric disorders was 18%, which is consistent with previous reports of hallucinations in nonclinical populations [44, 45]. Hallucinations among healthy people without apparent neuropsychiatric manifestations occur in a variety of conditions, including isolated sleep paralysis, migraine, sensory deprivation, Charles Bonnet syndrome, and preclinical psychosis [17, 18, 46, 47]. A control participant reported feelings of pushing in the chest with vestibulomotor hallucinations during a hypnopompic immobile state. This experience was considered isolated sleep paralysis. Tactile and vestibular hallucinations are common in isolated sleep paralysis [43]. Some controls reported hallucinations of deceased family or friends, which seemed to be postbereavement hallucinations (grief hallucinations) [48]. The prevalence of postbereavement hallucinations has been reported to range from 30 to 80% [45, 46]. Unless researchers are attentive, the prevalence is usually underestimated due to recall bias, fear of stigma, and negative connotation of the word hallucination [49]. In this study, we did not ask whether the participants had postbereavement experiences.

Several limitations should be mentioned. First, our study used an original questionnaire for screening hallucinations and their timing of occurrence. Therefore, only subjective phenomena were reported and analyzed. Objective assessments can be conducted with multiple sleep latency tests for sleepiness and wearable sensors, electroencephalograms or polysomnography during sleep [50]. Further investigation combining interviews with neurophysiological studies is warranted. Second, our study employed the term “sleep-related hallucinations”, which did not distinguish between hypnagogic and hypnopompic hallucinations as in previous studies [10, 15]. The difference between hypnagogic and hypnopompic hallucinations also needs to be characterized in further studies. Third, we did not evaluate the age of onset of hallucinations, and the frequency of hallucinations was not investigated. Fourth, hallucinatory events among healthy adults seem to have various origins, including isolated sleep paralysis, postbereavement hallucinations, poor vision due to aging and others. It would be inadequate to simply compare hallucinations in PD patients with those in a composite population. Fifth, our study did not focus on postbereavement hallucinations and their influence was underestimated. Sixth, our study lacked sleep diaries or actigraphs, and the profiles of the sleep-wake cycle of individuals were not examined. Seventh, we did not perform the Stanford Sleepiness Scale, which evaluates sleepiness throughout the day. We performed only a momentary assessment of sleepiness. Finally, this was a cross-sectional observational study. Therefore, the causality between the investigated factors and sleep-related hallucinations remains uncertain.

## Conclusions

Sleep-related hallucinations, but not daytime hallucinations, are associated with treatment duration and probable RBD. Phenomenological discrimination between sleep-related hallucinations and daytime hallucinations is important for elucidating the full pathology in Parkinson’s disease and the mechanisms underlying hallucinations.

## Supporting information

S1 File Original questionnaire for screening hallucinations. (PDF)

S2 File The excel file of the dataset. (XLSX)
