# Supplementary material for: Sleep-related hallucinations in patients with Parkinson’s disease
Source: PLoS One. 2022 Oct 25;17(10):e0276736. doi: 10.1371/journal.pone.0276736 (PMC9595548; doi:10.1371/journal.pone.0276736)
Supplement: S1 File — (PDF) [file pone.0276736.s001.pdf]

Questionnaire

We are investigating hallucinations experienced by Parkinson's disease patients and healthy individuals. Hallucinations are feelings or perceptions without existence or actual stimulus that can involve any sensory modality. Patients with Parkinson's disease often have these hallucinations. However, further study is needed to evaluate the physiological origin, sensitivity, or treatment of the phenomena. We intend to use the results of this study for the development of medical care and research in the future. If you agree to participate in the study, please answer all the questions below as accurately as possible.

Do you ever experience distorted vision in which straight lines appear curved or see colours or patterns that you should not?

□ Yes □ No

Please specify:

Do you ever see things that should not be there or people who should not be there (hallucinations)?

□ Yes □ No

Please specify:

When do you have such sensory experiences?

□ When wide awake during daytime activities □ Around sleep □ Both

Do you feel like someone is there even though you cannot see them?

□ Yes □ No

When do you have such sensory experiences?

□ When wide awake during daytime activities □ Around sleep □ Both

Do you hear sounds or voices that you should not hear (music, footsteps, voices, etc.)?

□ Yes □ No

Please specify:

When do you hear such sounds?

□ When wide awake during daytime activities □ Around sleep □ Both

Do you experience anything similar to the sensation of touch, such as being touched with something gritty or cool or being licked?

□ Yes □ No

Please specify:

When do you have such experiences?

□ When wide awake during daytime activities □ Around sleep □ Both

Do you smell an odour when there should not be an odour, or is there something that has been odorous for a long time?

□ Yes □ No

Please specify:

When do you have such experiences?

□ When wide awake during daytime activities □ Around sleep □ Both

Thank you for your cooperation.
